# Feasibility and effects of cognitive training on cognition and psychosocial function in Huntington’s disease: a randomised pilot trial

**DOI:** 10.1007/s00415-024-12855-7

**Published:** 2025-01-23

**Authors:** Katharine Huynh, Sharna D. Jamadar, Amit Lampit, M. Navyaan Siddiqui, Julie C. Stout, Nellie Georgiou-Karistianis

**Affiliations:** 1 Turner Institute for Brain and Mental Health, School of Psychological Sciences, Faculty of Medicine, Nursing and Health Sciences, Monash University, 18 Innovation Walk, Clayton, Victoria 3800 Australia; 2 Academic Unit for Psychiatry of Old Age, Department of Psychiatry, The University of Melbourne, Grattan St, Parkville, Victoria 3010 Australia; 3 Monash Biomedical Imaging, Monash University, 770 Blackburn Rd, Clayton, Victoria 3800 Australia

**Keywords:** Cognitive training, Huntington disease, Pilot trial, Cognition

## Abstract

**Background:**

Huntington’s disease (HD) is a rare neurodegenerative disease that causes progressive cognitive, physical, and psychiatric symptoms. Computerised cognitive training (CCT) is a novel intervention that aims to improve and maintain cognitive functions through repeated practice. The effects of CCT have yet to be established in HD. This randomised pilot trial examined the feasibility of a large scale trial to assess efficacy of multidomain CCT in pre-manifest and early-stage HD.

**Methods:**

28 participants were randomised to either at-home CCT (2 × 60 min sessions per week for 12 weeks; *n* = 13) or lifestyle education through monthly newsletters* (n* = 15). Participants completed cognitive tasks and questionnaires at baseline and follow up, either in person (*n* = 18) or via video teleconferencing (*n* = 10).

**Results:**

All participants were retained at follow up, and adherence to CCT ranged from 96 to 100%, with 11/13 participants completing all sessions. Preliminary analyses showed evidence of a large effect of CCT on task switching and response inhibition, compared to lifestyle education. There was no evidence of specific benefit to other cognitive domains (processing speed, basic and divided attention, working memory), or psychosocial functions (subjective cognition, mood, health-related quality of life).

**Discussion:**

Whilst retention and adherence rates were high, recruitment rates were low, suggesting that a large scale trial may be feasible with some modifications to increase recruitment rates, such as by reducing time burden associated with the study, and using a multi-site trial design. Potential effects on cognitive functioning warrant further investigation.

**Clinical trial registration**: The trial was prospectively registered on the Australian New Zealand Clinical Trials Registry (ACTRN12622000908730).

**Supplementary Information:**

The online version contains supplementary material available at 10.1007/s00415-024-12855-7.

## Introduction

Huntington’s disease (HD) is a rare, genetically inherited neurodegenerative disease that leads to cognitive, motor and psychiatric symptoms [1]. Clinical diagnosis is based on characteristic motor symptoms such as chorea [2], and occurs on average around 40 years of age [3]. Commonly, cognitive symptoms often occur prior to diagnosis in the pre-manifest stage [4, 5], with decline in processing speed, cognitive flexibility, attention, and working memory [4–8]. Despite the impact of cognitive symptoms, there are no established treatments to delay cognitive decline [3, 9, 10].

Cognitive training aims to improve cognitive functions through repeated practice of tasks targeting those functions [11]. Computerised cognitive training (CCT) is completed on a digital device such as a computer or tablet [12]. Cognitive training has been shown to improve cognition in healthy older adults and clinical populations such as Parkinson’s disease and multiple sclerosis [13–18]. Proposed mechanisms of cognitive training include structural and functional brain changes that increase the capacity and efficiency of neural networks [19–23]. There is also evidence of benefits to psychosocial functioning, including subjective cognition, mood, and quality of life, in healthy older adults [15] and individuals with mild cognitive impairment [24]. Whilst mechanisms for benefits to psychosocial functioning are unclear, studies have suggested indirect benefits to psychosocial functioning through improvements in cognition [25, 26], as well as placebo effects associated with active engagement in the intervention [27].

Research into the effects of cognitive training in HD is still preliminary. There have been three existing studies of cognitive training in HD (two single arm studies and one randomised feasibility trial), which together have provided low-quality evidence of a small positive effect on cognition [28]. The recent randomised feasibility trial of executive function training [29] deemed the intervention to be unfeasible due to low adherence: 4/10 participants completed the intervention. Barriers to adherence included repetitiveness of exercises, lack of time (the intervention required 3 × 30 min sessions per week), and impact of disease stage (people with middle-to-late-stage HD were also recruited).

To address limitations and barriers to adherence noted in previous studies, we conducted a randomised pilot trial in individuals with pre-manifest and early-stage HD, comparing a multidomain CCT intervention (2 × 60 min sessions per week, with one weekly supervised session) with lifestyle education, the current gold standard in preventing cognitive decline. We assessed feasibility outcomes such as recruitment rates, adherence, and retention. We assessed effects of CCT on (1) processing speed, the primary cognitive outcome; (2) secondary cognitive outcomes, including attention, working memory, inhibition, and cognitive flexibility; and (3) secondary psychosocial outcomes, such as subjective cognition, mood, and health-related quality of life. We hypothesised improvements from baseline to follow up in the CCT group, compared to the lifestyle education group.

## Methods

The trial was prospectively registered on the Australian New Zealand Clinical Trials Registry (ACTRN12622000908730). The protocol was approved by the Monash University Human Research Ethics Committee. Informed consent was obtained from participants via a written consent form. The protocol has been published previously [30]. As such, the methods are briefly summarised here. Deviations from the published protocol are elaborated upon.

### Trial design

The study had a parallel arm randomised controlled design, with 1:1 allocation ratio to either intervention (CCT) or control (lifestyle education).

### Participants

Participants were initially recruited within Melbourne, Australia by screening an existing HD research database and referral of people with HD by clinicians at hospitals across Melbourne. However, following challenges with local recruitment, recruitment was expanded across Australia by allowing individuals to complete supervised remote assessments (see ‘Study Procedure’ section).

People were considered eligible if they were: (a) HD gene-positive (CAG repeat length > 35), (b) in either pre-manifest or early manifest stage of HD, as defined by having a Total Functional Capacity (TFC) score of 7 or above on the Unified Huntington’s Disease Rating Scale (UHDRS), and (c) had access to a computer with internet connection. People were excluded if they were (a) under 18 years of age, (b) had a diagnosis of any major neurological or psychiatric conditions other than HD, (c) had a history of substance abuse or head injury, (d) currently experiencing severe anxiety or depression symptoms, as defined by a score of > 14 on either subscale of Hospital Anxiety and Depression Scale [HADS]), or (e) had changes in their medication for anxiety or depression in the last 6 months.

### Interventions

#### Intervention: computerised cognitive training

Participants in the CCT group completed 60-min CCT sessions, twice a week, for 12 weeks on a computer within their own homes. One session each week was supervised remotely by a researcher through video teleconferencing, as supervision is associated with increased adherence and increased efficacy [31–33]. Dosing and supervision methods were established based on published recommendations, which suggest greater benefit for training sessions that are longer (> 30 min) and less frequent (no more than three sessions per week) [31, 34], as well as participant feedback in previous feasibility studies of CCT in HD [29]. A regular training schedule (day and time of training) was established with a researcher during the first supervised training session to support adherence. CCT was conducted using BrainHQ [35], which has demonstrated efficacy in other populations [36, 37]. The training schedule involved visual and auditory tasks that target processing speed, attention, working memory, inhibition, and cognitive flexibility. The programme was adaptive, such that the difficulty level adjusted to user performance. The selection of cognitive exercises is described in Supplementary Material (Table S1**).**

#### Control: lifestyle education

The control group received lifestyle education through three monthly newsletters focusing on physical activity, cognitive and social engagement, and diet. Lifestyle education is considered the current ‘gold standard’ to support cognition in people with HD, and there are no apparent differences in efficacy of CCT depending on whether control group is active (e.g. non-adaptive training) or passive (e.g. waitlist control) [31, 36]. Lifestyle education group participants received monthly check-in calls. During these calls, participants provided feedback on the newsletters and reported any lifestyle changes following the newsletters, although they were not requested to make lifestyle modifications. Lifestyle education group participants also received access to CCT for 12 weeks after study participation.

### Study procedure

**Fig. 1:**
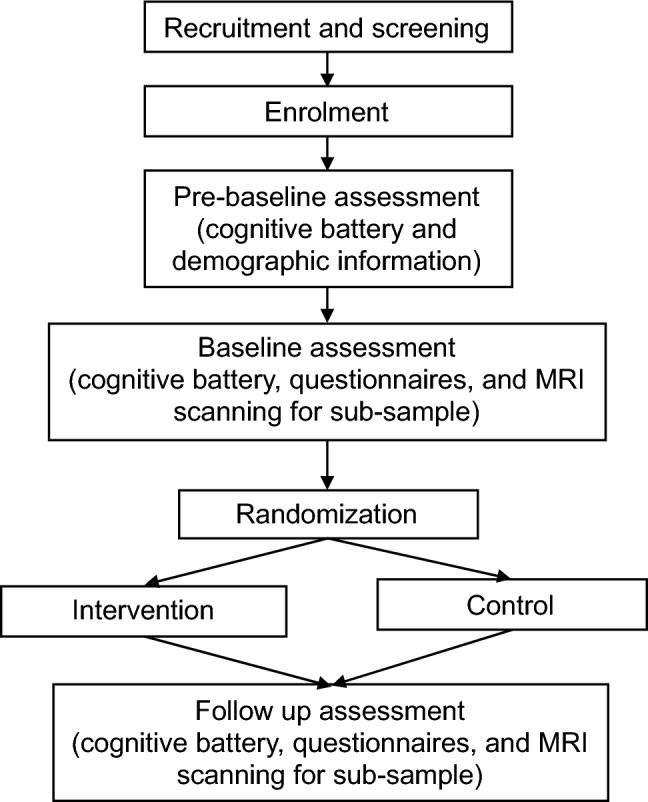
Flow chart depicting timeline of enrolment, assessments, and randomisation of participants

Following enrolment, participants completed assessments at three timepoints: pre-baseline, baseline, and follow up (Fig. 1). Assessment sessions were completed either in person or remotely via video teleconferencing, depending on the residential location of the participant. In-person sessions were conducted either at participant homes or at Monash Biomedical Imaging in Clayton, Australia. For remote assessments, participants were mailed forms for the assessment in a secure envelope and returned the forms via mail.

The pre-baseline assessment involved cognitive testing to reduce practice effects, and collection of relevant demographic information. Premorbid intelligence was estimated using the National Adult Reading Test (NART) [38]. Following relaxation of handedness criterion for MRI scanning, handedness was assessed using the Edinburgh Handedness Inventory (EHI) [39]. The UHDRS motor examination was conducted by a trained researcher if the UHDRS Total Motor Score (TMS) was not available from the participant’s HD clinician and the assessment was in-person. Baseline and follow up assessments involved cognitive tests and questionnaires. A subset of participants completed MRI scans following the cognitive assessment; results of which are not reported here. At each timepoint, assessments were conducted by researchers blinded to the allocation of the participant.

### Randomisation

Following baseline assessment, participants were randomised to a group using minimisation with a biased coin method, with MinimPy2 software [40]. Minimisation was used to balance the following variables between groups: age, sex, years of education, disease stage (TFC score), severity of anxiety/depression symptoms (HADS score), consent to MRI. Allocation was conducted by an off-site researcher not involved in recruitment or assessments.

### Feasibility outcomes

Feasibility outcomes included rates of recruitment, retention, and adherence to intervention. Adherence to intervention was assessed according to proportion of target training sessions completed, and improvement in training exercises (based on percentile rankings provided by the BrainHQ program).

### Cognitive and psychosocial outcomes

The primary cognitive outcome was processing speed, according to the score on Symbol Digits Modalities Test (SDMT) [41]. The SDMT was chosen as the primary outcome measure as it is sensitive to cognitive deficits in pre-manifest stages of the disease, and shows large effect sizes in early-stage HD compared to healthy controls [7, 42]. It is predictive of disease progression, including time to diagnosis and decline in functional capacity after diagnosis, even after controlling for age and CAG length [5, 43].

Secondary cognitive outcomes included simple processing speed (total correct on Stroop word and colour conditions [44]), attention (time to complete Trail Making Test A [45], cognitive flexibility (time to complete Trail Making Test B [45], Trails B efficiency score [Trails Be] [46]), verbal working memory (total score on Digit Span [47]), visuospatial working memory (total score on Spatial Span [47]), and inhibition (total score on Stroop interference [44]). The Trails B efficiency score (Trails Be), derived from measures of completion time and errors, was introduced as an outcome measure after trial commencement to more effectively analyse performances below the test floor (exceeding maximal time limit) [46].

Cognitive flexibility and processing speed were also assessed using two computerised experimental tasks: a letter-number task switching paradigm (TSWT) [48] and a modified SDMT [49], respectively. Details of experimental tasks are provided in Supplementary Material. In brief, the TSWT paradigm involves participants switching randomly between completing either a letter task or a number task. Derived outcome measures include average accuracy and reaction times on switch and repeat trials, and switch costs (difference in performance between switch and repeat trials). The modified SDMT requires indicating whether a digit-symbol probe matches a digit-symbol pair in a coding table. Derived outcome measures include average accuracy and reaction time. Computerised tasks were completed during an MRI scan or on a computer if the participant did not consent to MRI scanning.

Secondary psychosocial outcomes included subjective cognitive function (total score on Cognitive Difficulties Scale [50]), mood (total score on HADS [51]), and health-related quality of life (total score on HD-PRO-TRIAD [52]).

### Statistical analyses

Statistical analyses were conducted using RStudio (2023.9.1.494) [53]. Descriptive statistics were used to summarise baseline demographic and clinical variables, outcome variables for each group per timepoint, and feasibility outcomes. Within the training group, improvement on BrainHQ tasks was assessed using a repeated measures *t*-test.

Groups were compared at baseline on demographic (age, years of education, premorbid intellectual functioning [NART error score], handedness [EHI laterality index]), clinical variables (CAG repeat length, TFC score, disease burden score [DBS] [54], total HADS score), and cognitive and psychosocial outcomes using an independent samples *t*-test. Groups were also compared on distribution of sexes and handedness using a Chi-squared test.

Differences in change between groups from baseline to follow up on cognitive and psychosocial outcomes were assessed by calculating effect size (Cohen’s *d*) and 95% confidence intervals (CIs) using formula by Skvarc & Fuller-Tyszkiewicz (2024) [55]. Linear mixed effects (LME) models were used to test for Group x Time interaction effects, with significance level set at *p* < .05. In instances of discrepancies between effect size calculations and LME models, the more conservative outcome was considered in our interpretation. Significant interaction effects were examined post-hoc with repeated-measures *t*-tests and within-group effect size calculations (Cohen’s *d)* using formula by Cohen (1998) [56].

Sensitivity analyses included modality of assessment (face-to-face or remote) as a covariate in LME models. As experimental tasks (task switching and modified SDMT) were completed either in the MRI scanner or on a computer, the task context was also added as a covariate.

As this was a feasibility study, the sample was under-powered for analyses of efficacy. Post-hoc power calculations have been provided using G*Power [57], based on the acquired sample size and effect sizes reported in meta-analyses of CCT in other populations, with *α* = .05 [15].

## Results

The results of this pilot trial have been reported according to the CONSORT guidelines for pilot and feasibility trials [58].

### Participant flow

**Fig. 2:**
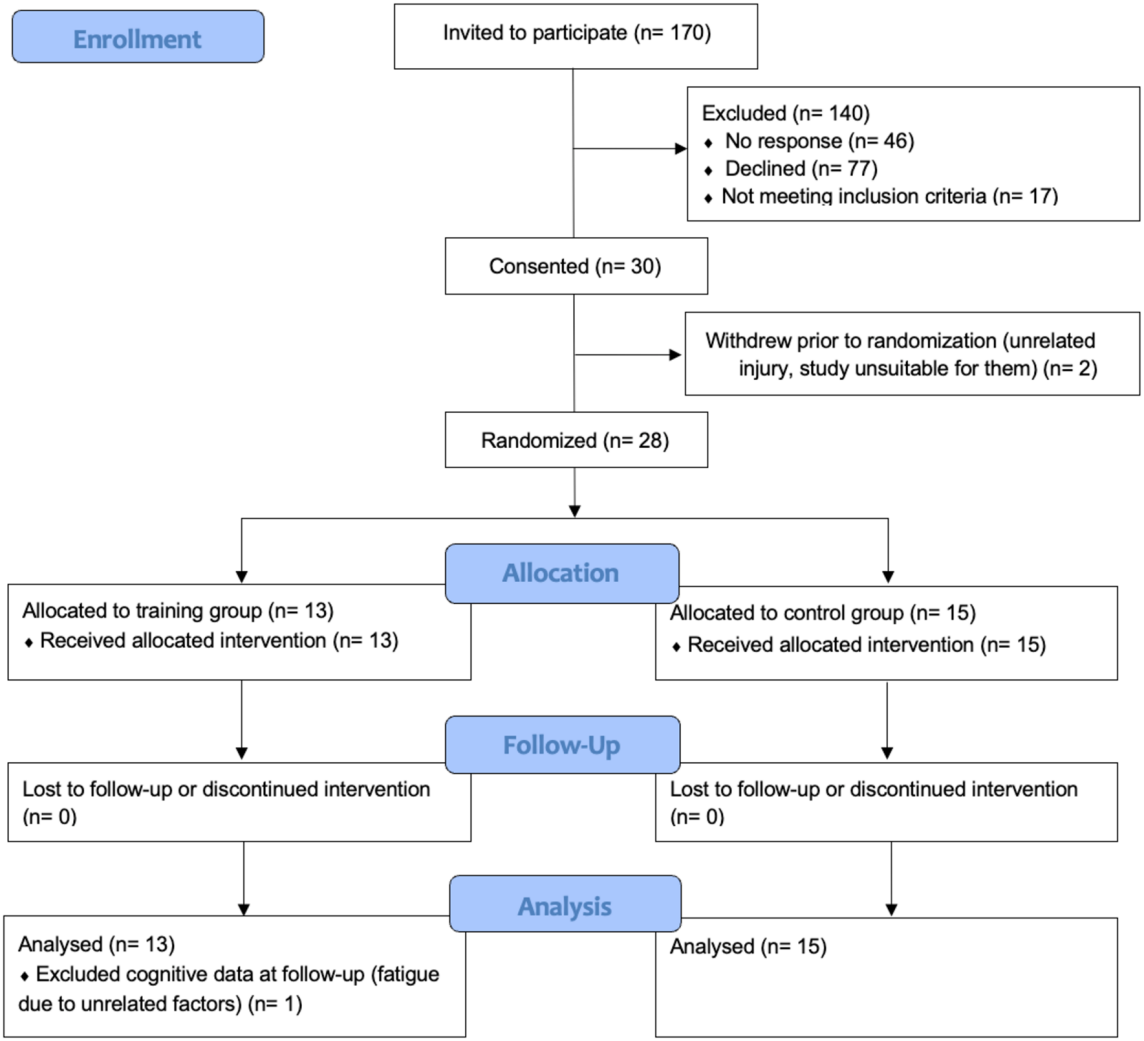
Participant CONSORT flow diagram

Recruitment occurred between June 2022 and December 2023 (18-month period). Participant recruitment and study flow are depicted in Fig. 2. After preliminary screening of the database and referrals from clinicians, 170 individuals were invited to participate in the study. Forty-six individuals did not respond, and 77 individuals declined the invitation. Amongst the 77 individuals who declined to participate, 39 individuals provided reasons for declining. This included: psychosocial factors including health concerns within the family or self (*n* = 15), lack of time/high study demands (*n* = 12), disease stage being too advanced (*n* = 5), concurrent participation in drug trial (*n* = 3) and lack of access/experience with computers (*n* = 2).

Seventeen individuals expressed interest but did not meet inclusion criteria following comprehensive screening. Thirty participants provided consent (28% of potentially eligible individuals who responded to the invitation). As such, we did not meet the recruitment target of *n* = 50 participants. Two participants withdrew prior to baseline and randomisation: one reported an unrelated injury and the other reported that the study was too demanding. Altogether, 28 participants were randomised (*n* = 13 CCT, *n* = 15 lifestyle education). Eighteen participants (7 CCT, 11 lifestyle education) completed assessment sessions in person and ten participants (6 CCT, 4 lifestyle education) completed assessments remotely. All randomised participants completed follow up assessments. One participant’s cognitive data at follow up was excluded from analyses due to self-report of significant fatigue due to non-study-related factors, and evidence of extreme values of change.

### Participant characteristics

Participant characteristics at baseline are presented in Table 1. No significant differences between groups were found in demographic characteristics, including age, sex, handedness, education level, or premorbid functioning. No significant differences between groups were found in clinical characteristics, including CAG repeat length, DBS, UHDRS TFC score, UHDRS TMS score, or HADS total score. Due to changes to the protocol, including introduction of EHI and remote assessments, EHI and UHDRS TMS scores are only available for 13/28 (6 training, 7 control) and 19/28 (8 training, 11 control) participants, respectively.

**Table 1:** Demographic and clinical characteristics of participants at baseline

Variable	Training (*n* = 13)	Control (*n* = 15)	Statistic (*p*-value)
Age (years)	53.85 (11.42)	50.53 (12.43)	.47
Sex (% male)	15.38%	40.00%	.31
Handedness (% right)	100.00%	86.67%	.53
Handedness (EHI laterality index)^a^	96.29 (5.77)	51.43 (84.74)	.21
Education (years)	17.12 (4.02)	15.40 (3.94)	.27
Premorbid functioning (NART error score)	16.15 (6.38)	18.87 (5.64)	.25
*CAG* repeat length	40.08 (2.18)	41.33 (1.99)	.13
DBS	243.31 (130.00)	296.67 (120.63)	.27
UHDRS TFC score	12.00 (1.87)	11.93 (2.02)	.93
UHDRS TMS^b^	8.00 (19.04)	5.36 (8.44)	.72
HADS total score	4.54 (4.18)	6.33 (4.94)	.31

*Note*: All summary characteristics are presented as mean (SD) unless otherwise stated. *p*-value is based on independent samples *t*-test for continuous variables, and Chi-squared test for categorical variables. Missing data are indicated with superscripts

*DBS* Disease burden score, *EHI* Edinburgh Handedness Inventory, *HADS* Hospital Anxiety and Depression Scale, *NART* National Adult Reading Test, *TFC* Total Functional Capacity, *TMS* Total Motor Score, *UHDRS* Unified Huntington’s Disease Rating Scale

^a^Data available only for *n* = 13 participants (6 training, 7 control)

^b^Data available only for *n* = 19 participants (8 training, 11 control)

### Feasibility outcomes

#### Rates of assessment completion

Due to practical reasons, only participants completing face-to-face assessments (18/28 participants [7 training, 11 lifestyle intervention]) were administered Spatial Span. All other administered cognitive tasks and questionnaires were successfully completed by all participants, except for the computerised experimental tasks (letter-number task switching and modified SDMT), which a small number of participants found too difficult. These were completed by 23/28 participants (10 training, 13 lifestyle education) and 27/28 (12 training, 15 lifestyle education) participants, respectively.

#### Retention and adherence

All participants randomised to either CCT group or lifestyle education group completed follow up assessments. Within the CCT group, adherence ranged from 96 to 100% of the target 24 sessions, with 11/13 participants completing 100% of sessions. Percentile improvement across all trained tasks averaged 18.92% (*SD* = 4.72), *t*(12) = 14.47, *p* < 0.001. 3/13 participants requested a break during the training period (1–2 weeks) due to scheduled holidays. One participant used a tablet to complete the CCT intervention due to significant chorea limiting keyboard and mouse use. Another participant reported similar difficulties with keyboard and mouse use, however, was unable to access a tablet.

#### Acceptability of intervention

Whilst acceptability data (e.g. thoughts on ease of use, intent to continue training) were not collected in a systematic manner, the training duration or frequency were not reported to be burdensome by participants in the training group. Furthermore, two of 13 participants in the CCT group reported a desire to continue with the intervention after the study. Several participants disclosed that the training was cognitively fatiguing, particularly with greater repetition of exercises that the participant found challenging, and occasional technological (Internet) difficulties were reported to be frustrating.

### Analyses of cognitive and psychosocial outcomes

Statistical analyses were conducted to explore potential effects of CCT in HD and identify sensitive outcome measures for further investigation, rather than evaluate efficacy of CCT. Post-hoc power analyses suggested that with a sample size of *n* = 28, the power to detect a small effect size (*d* = 0.40, which is reported in previous meta-analyses of CCT in other populations [15]) is 17%. As such, results described below should be interpreted with caution. Effect sizes and CIs are provided to demonstrate the imprecision of the effect size estimate.

There were no statistically significant differences between groups in cognitive or psychosocial outcomes at baseline (*p*’s > 0.05). Table 2 illustrates descriptive statistics for cognitive and psychosocial outcomes at baseline and follow up, and inferential statistics including effect size estimates with 95% CI comparing changes from baseline to follow up between groups, and LME model analyses for Time x Group interaction effects. Across outcomes, effect size estimates ranged from large and positive (representing benefit of CCT compared to lifestyle education) to large and negative (representing decrement of CCT compared to lifestyle education). Across all outcomes, there were wide CIs, suggesting imprecision of estimates. Sensitivity analyses that included modality of assessment and experimental task context as covariates in LME models did not change patterns of statistical significance (Supplementary Material).

**Table 2:** Descriptive data and inferential statistics for cognitive and psychosocial outcomes

Variable	Intervention (*n* = 13)			Control (*n* = 15)			Statistics		
	*N*^a^	T1	T2	*N*	T1	T2	Cohen’s *d* (95% CI)	*F*	***p***
SDMT	12	57.92 (23.34)	53.08 (22.72)	15	48.07 (16.77)	48.47 (17.86)	**−0.94 [−1.74, −0.14]**	4.19	0.05
Digit Span Forwards	12	10.54 (2.40)	10.50 (3.03)	15	10.67 (2.61)	10.13 (2.26)	0.28 [−0.48, 1.05]	0.53	0.47
Digit Span Backwards	12	7.46 (2.93)	7.33 (3.11)	15	6.47 (2.20)	6.60 (2.17)	−0.16 [−0.92, 0.60]	0.07	0.80
Stroop Colour	12	72.76 (24.94)	76.33 (25.98)	15	71.93 (22.38)	69.87 (20.96)	**0.86 [0.07, 1.65]**	3.64	0.07
Stroop Word	12	87.46 (29.13)	91.08 (29.45)	15	88.53 (25.18)	87.40 (24.19)	0.68 [−0.10, 1.46]	4.07	0.05
Stroop Interference	12	45.15 (17.48)	48.08 (21.42)	15	45.40 (16.25)	42.53 (15.15)	**0.96 [0.16, 1.76]**	11.69	0.002**
Trails A	12	26.31 (23.12)	30.67 (27.52)	15	25.33 (15.26)	25.00 (17.03)	−0.70 [−1.48, 0.08]	2.68	0.11
Trails B	12	71.62 (70.23)	76.75 (82.52)	15	66.93 (50.52)	59.80 (42.62)	−0.59 [−1.37, 0.18]	1.70	0.20
Trails Be	12	3.10 (3.00)	3.61 (4.32)	15	2.97 (2.33)	2.54 (1.86)	−0.63 [−1.41, 0.15]	2.97	0.10
Spatial Span Forwards	6	8.86 (2.48)	9.00 (2.10)	11	9.00 (2.00)	8.09 (1.76)	0.65 [−0.37, 1.67]	2.02	0.18
Spatial Span Backwards	6	8.43 (2.15)	8.50 (2.95)	11	7.73 (1.68)	7.46 (2.25)	0.16 [−0.83, 1.15]	0.04	0.84
TSWT Switch Accuracy (%)	9	95.07 (5.11)	97.36 (2.03)	13	95.99 (2.87)	93.82 (3.84)	**1.01 [0.11, 1.91]**	7.40	0.01*
TSWT Switch Reaction Time (ms)	9	990.37 (235.21)	944.96 (223.49)	13	1004.41 (190.43)	1004.81 (252.99)	0.26 [−0.59, 1.11]	0.75	0.40
TSWT Repeat Accuracy	9	97.75 (2.32)	97.03 (3.11)	13	96.64 (2.48)	96.42 (3.57)	−0.18 [−1.03, 0.67]	0.15	0.71
TSWT Repeat Reaction Time (ms)	9	965.42 (233.19)	937.06 (241.65)	13	948.61 (199.91)	962.32 (230.50)	0.26 [−0.59, 1.12]	0.72	0.41
TSWT Switch Cost (Accuracy, %)	9	2.68 (3.28)	−0.33 (2.33)	13	0.65 (2.91)	2.60 (2.43)	**1.13 [0.21, 2.04]**	8.84	0.005**
TSWT Switch Cost Reaction Time (ms)	9	24.95 (45.83)	7.901 (90.36)	13	55.81 (44.17)	42.50 (66.87)	0.04 [−0.81, 0.89]	0.01	0.92
mSDMT Accuracy (%)	11	92.07 (11.12)	94.89 (4.05)	15	90.70 (10.63)	92.59 (7.50)	0.13 [−0.65, 0.91]	0.20	0.66
mSDMT Reaction Time (ms)	11	1530.59 (274.35)	1471.30 (347.06)	15	1632.72 (393.62)	1662.70 (388.68)	0.74 [−0.06, 1.54]	3.70	0.07
CDS	13	77.31 (32.27)	69.85 (32.69)	15	85.87 (29.65)	67.27 (24.76)	−**1.02 [**−**1.74, −0.14]**	2.20	0.15
HADS	13	4.54 (4.18)	4.00 (4.80)	15	6.33 (4.94)	6.67 (5.72)	0.25 [−0.48, 1.05]	0.45	0.51
HD-PRO-TRIAD	13	4.55 (1.40)	4.44 (1.68)	15	5.51 (2.03)	4.93 (2.11)	−0.47 [−0.92, 0.60]	1.77	0.19

*Note:* Data are presented as mean (SD) unless otherwise stated. Bolded effect size estimates and *p*-values are considered statistically significant

*T1* baseline, *T2* follow up, *SDMT* symbol digits modalities test, *TSWT* task switching, *mSDMT* modified SDMT, *CDS* cognitive difficulties scale, *HADS* Hospital Anxiety and Depression Scale

^a^Cognitive data at follow up was removed for 1 participant. *N* is representative of number of observations at follow up

#### Cognitive outcomes

**Fig. 3:**
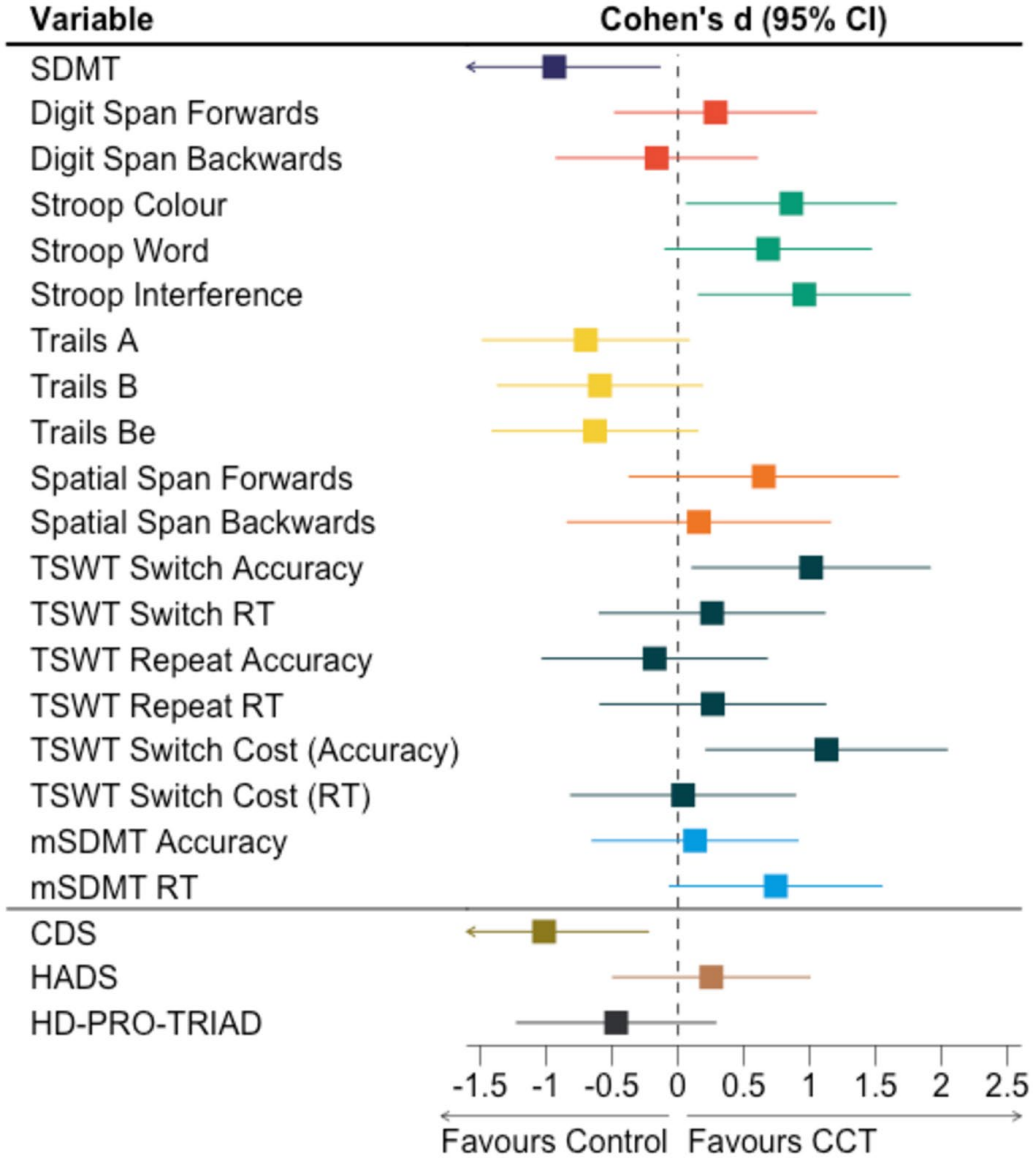
Effect sizes (Cohen’s *d*) and 95% confidence intervals for cognitive outcomes (above border) and psychosocial outcomes (below border). Larger positive effect sizes represent greater benefit of cognitive training intervention compared to lifestyle education. *CCT* computerised cognitive training, *CI* confidence interval, *SDMT* symbol digits modalities test, *TSWT* task switching, *mSDMT* modified SDMT, *RT* reaction time, *CDS* Cognitive Difficulties Scale, *HADS* Hospital Anxiety and Depression Scale

**Fig. 4:**
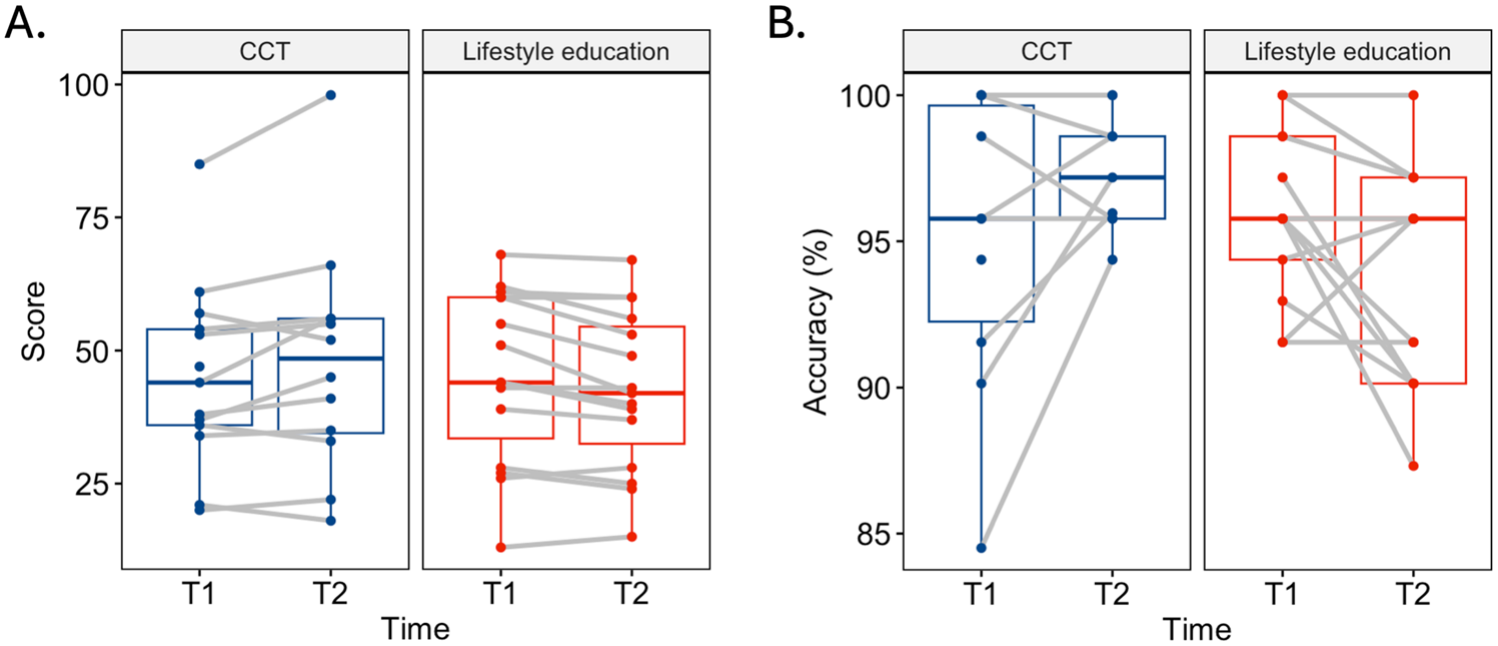
Change in performance on Stroop interference score (**A**) and task-switching switch accuracy (**B**) from baseline to follow up. Higher scores represent better performance *CCT* computerised cognitive training, *T1* baseline, *T2* follow up

For majority of cognitive outcome measures (Digit Span, Stroop Word condition, Trails A and B, Spatial Span, and modified SDMT), there was no evidence of effect of CCT compared to lifestyle education (i.e. 95% CIs for effect size calculations included 0, and *p*’s > 0.05 in LME models; Fig. 3; Table 2). For Stroop interference, TSWT switch accuracy and switch cost accuracy measures, there was evidence of large positive effect of CCT compared to lifestyle education (i.e. 95% CI excluded 0*, p*’s < 0.05 in LME models). TSWT switch cost accuracy is derived from differences between switch trial accuracy and repeat trial accuracy. Considering evidence of benefit to TSWT switch accuracy but not TSWT repeat accuracy, we interpreted changes in switch cost accuracy as driven by changes in switch trial accuracy. Post-hoc within-group analyses revealed significant declines in the lifestyle education group (with small-to-moderate effect sizes) on Stroop interference (*t*(14) = −3.37, *p* = 0.005, *d* = −0.17 [−0.27, −0.07]), and TSWT switch accuracy (*t*(12) = −2.22, *p* = 0.005, *d* = −0.63 [−1.24, −0.02]). In contrast, there were no significant changes in the CCT group on these outcome measures (*p’s* > 0.05, 95% CIs included 0). Vizualisation of individual values at baseline and follow up showed heterogeneity in change within the CCT group for both outcome measures (Fig. 4). Qualitatively, factors such as disease stage, age, and CAG length did not appear to coincide with differences in individual change (Supplementary Material, Fig. S3).

Discrepancies between effect size calculations and LME models were noted for Stroop Colour and SDMT performance. Specifically, effect size calculations showed evidence of large positive effect of CCT compared to lifestyle education on Stroop Colour performance (*d* = 0.86 [0.07, 1.65]) and large negative effect on SDMT performance (*d* = −0.94 [−1.74, −0.14]). However, LME models did not demonstrate significant interaction effects (*p*’s > 0.05). Given the small sample and wide CIs which approach 0, the more conservative LME results were favoured in our interpretation.

#### Psychosocial outcomes

Effect size calculations showed a large negative effect of CCT compared to lifestyle education on CDS (subjective cognition) scores (Fig. 3; Table 2). However, the LME model showed no significant interaction effect. Instead, there was a significant main effect of timepoint (*p* = 0.008). Examination of within-group change revealed a small, significant improvement in CDS in the CCT group (*t*(12) = 3.42, *p* = 0.005, *d* = 0.23 [0.10, 0.36], and a medium, significant improvement in CDS in the lifestyle education group (*t*(15) = 3.63, *p* = 0.003, *d* = 0.51 [0.22, 0.80]). There was no evidence of effect of CCT on HADS (mood) or HD-PRO-TRIAD (health-related quality of life) scores, compared to lifestyle education (95% CI included 0, *p*’s > 0.05 in LME models).

## Discussion

This pilot trial assessed the feasibility of a large scale randomised controlled trial (RCT) to examine the effects of a partially supervised, multidomain CCT intervention in pre-manifest and early-stage HD. Participants were assessed either in person or via video teleconferencing. The study provides evidence of high retention and adherence, however the recruitment target was not met. Analyses showed preliminary evidence of positive effects on executive domains (task switching and response inhibition), with no evidence of specific benefits to psychosocial functions. As such, further investigation of the efficacy of CCT on cognition is warranted. A large scale RCT may be implemented with some modifications to the current protocol to facilitate recruitment.

### Feasibility

We obtained high retention and adherence to our CCT intervention, suggesting that the trial and intervention design may be acceptable for individuals in pre-manifest and early stages of HD. Notably, our retention and adherence rates were greater than those obtained in a previous feasibility study of a 3-month executive functioning CCT intervention. The greater retention and adherence obtained here may be attributable to the multidomain nature of training (reducing repetitiveness of exercises), weekly supervised sessions that increase engagement, and recruitment of participants in earlier stages of HD. It is also possible that there was sampling bias that led to greater engagement with the study.

Despite high retention and adherence rates, there was a low recruitment rate (28% of individuals who responded to the invitation enrolled), and we did not achieve recruitment targets. This is similar to previous studies of CCT in HD [29, 59]. Study-related barriers included that the study was too time-intensive and demanding. In addition, lack of access to computers and computer literacy were barriers to participation for a small number of individuals. Notably, a greater proportion of individuals declined to participate due to non-study-related factors, such as psychosocial stress and poor health related to self or family, which may be associated with the inter-generational and biopsychosocial burdens of HD. In addition, despite introducing remote assessments to reduce participant burden and facilitate recruitment of individuals residing further away from the study site, the number of eligible individuals was limited, suggesting inherent recruitment challenges due to the low prevalence of HD.

In consideration of the low recruitment rate and the noted barriers to participation, our participants likely represent a subset of individuals who are ‘high functioning’, or have greater social support (typically family) to engage with research. It is likely that similar rates of adherence and retention may not be obtained in individuals with HD who are experiencing greater functional challenges or lack computer literacy, and do not have the social or environmental support to compensate for these factors. This suggests that the study may require modifications to increase recruitment rates in a large scale RCT (see *Implications* secton below).

### Effects on cognition and psychosocial function

Amongst cognitive outcomes, it appeared that cognitive flexibility (measured by TSWT switch accuracy) and response inhibition (measured by Stroop interference) abilities were modified by CCT. On average, performance was maintained in the CCT group, but declined in the lifestyle education group. These findings suggest CCT may benefit proactive cognitive control mechanisms [60], which facilitate maintenance of goal-relevant information and inhibition of task-irrelevant information, and is typically impacted by neurodegeneration in HD [61]. This finding is consistent with a recent meta-analysis in healthy older adults that found that executive functions are the most responsive cognitive domains to CCT, compared to domains such as memory and reasoning [18]. Similarly, another meta-analysis in healthy older adults reported a small, significant effect of multidomain CCT on cognitive flexibility and inhibitory control [62]. Neurologically, these cognitive domains are mediated by the lateral prefrontal cortex [60] and the middle frontal gyrus [63, 64]. Consistent with this, exploratory MRI analyses in a subset of our participants showed increased grey matter volumes in the right middle frontal gyrus following CCT, in contrast to decreased volumes in the lifestyle education group [65].

Decline in cognitive performance in the lifestyle education group over the short timeframe of our study (3 months) was unexpected. However, it is consistent with the finding that in the subset of participants who underwent MRI scanning, the lifestyle intervention group showed decreased grey matter volumes from baseline to follow up in several regions [65]. Our result contrasts with a previous study that reported significant decline in SDMT in pre-manifest and early-stage HD over a 15-month interval, but not shorter 6-month intervals [66]. Whilst it is unclear why the lifestyle intervention group showed reduced cognition over this period, the finding may be accounted for by several factors. First, previous studies of longitudinal change in HD typically compared change with healthy controls and did not adjust for practice effects [66], which may reduce sensitivity to detecting change [67]. Our within-group analyses may have been more sensitive to change because we did not compare changes with healthy controls, and accounted for practice effects using a pre-baseline session. In addition, it is possible that this finding was driven by sampling bias within our sample, given the substantial inter- and intra-individual variability in cognitive trajectories in HD [68].

Whilst performances on several cognitive measures were maintained on average in the CCT group, heterogeneity across participants was evident. Examining individual performance, it is evident that some participants improved after CCT, and some declined indicating variability in individual response to CCT [20]. This response variability may have contributed to the wide CI observed across outcome measures. Unfortunately, the small sample size precluded analysis of effect modifiers. However, qualitatively, factors such as disease stage, age, and CAG length did not appear to explain differences in individual change.

In contrast to hypotheses, there was no evidence of benefits of CCT to processing speed, attention, and working memory domains. The evidence for the effect of CCT on these domains in the literature is equivocal. The results partially align with two previous meta-analyses in healthy older adults, which found no significant effects of multidomain CCT on processing speed and attention [31, 62]. It is possible that these domains require a greater dose of single-domain training [62]. Interestingly, Lampit et al. (2014) [31] and Nguyen et al. (2019) [62] reported significant improvement in working memory following multidomain CCT in older adults without cognitive impairment. Differences in our findings may be due to differences in neuropathology across healthy ageing and neurodegenerative conditions. It may also be due to the heterogeneity in cognitive functioning in our sample, with greater cognitive impairment limiting capacity to improve working memory. Consistent with this, a meta-analysis of CCT in people with mild cognitive impairment found no significant improvements in working memory [69].

Regarding psychosocial functions, there was no evidence of greater benefit of CCT to subjective cognition, mood, and health-related quality of life, compared to lifestyle education. Whilst there was an improvement in subjective cognition following CCT, a greater improvement was found following lifestyle education. However, the improvement in subjective cognition in the lifestyle education group was likely driven by placebo effects rather than an accurate reflection of cognitive gains. Indeed, after reading each newsletter, participants in the lifestyle education group often reported feeling reassured about their existing healthy lifestyle choices, rather than taking up new lifestyle changes. Overall, these findings align with existing studies of cognitive training in HD, which do not show evidence of benefits to psychosocial functioning [28]. Similarly, meta-analyses of studies in other neurodegenerative conditions, such as Parkinson’s disease [70] and multiple sclerosis [17] report no significant effects of CCT on psychosocial outcomes such as mood and quality of life.

### Implications

This was a randomised pilot trial to examine feasibility of conducting a large scale trial of CCT in pre-manifest and early stage HD. As such, the study was inadequately powered for analyses of efficacy and the results should be taken as preliminary to explore potential effects of CCT. The effect size estimates are imprecise (demonstrated by wide CIs), and analyses were not corrected for multiple comparisons.

Whilst there was a high level of adherence and retention, recruitment rates were low due to reasons discussed previously. Therefore, participant engagement to co-design a desirable CCT intervention (in particular, establishing acceptable and efficacious training schedules), would be an important next step to promote participation in a large scale trial, as well as to increase uptake and implementation of CCT as a cognitive intervention. Importantly, the context of assessments (face-to-face or remote) did not significantly affect retention, adherence, or pattern of results. This is consistent with evidence of feasibility of remote neuropsychological testing in other neurodegenerative conditions such as Alzheimer’s disease and frontotemporal dementia [71]. Therefore, future trials may seek to increase recruitment rates by employing remote assessments to reduce participant burden and extend recruitment to individuals residing in rural areas [72], as well as by employing a multi-site trial design. When working with rare conditions such as HD, where recruitment and statistical power is often a challenge, efficacy of an intervention may also be assessed through meta-analyses of smaller trials [73].

Additional proposed amendments for future trials include intervention delivery on a tablet for participants with chorea to improve their experience and engagement with the intervention. We also recommend caution when scheduling assessments to limit exposure to test outcomes through other avenues (e.g. research or neuropsychological assessments).

Overall, our pilot trial suggests potential feasibility of a large scale RCT to assess efficacy of multidomain CCT in pre-manifest and early-stage HD, following some modifications to increase recruitment rates. Preliminary analyses showed evidence of maintenance of task switching and response inhibition following CCT, compared to lifestyle education. This is consistent with our finding of preserved/increased grey matter volumes in the CCT group, in the subset of participants who completed MRI [65]. There was no evidence of benefit to other cognitive domains (processing speed, basic and divided attention, working memory), or psychosocial functioning (subjective cognition, mood, health-related quality of life), compared to lifestyle education. This study contributes to a small yet growing body of evidence suggesting potential benefits of CCT to cognition in HD [28]. Larger multi-site trials or meta-analysis of smaller trials are warranted to evaluate the efficacy of CCT and explore effect modifiers such as disease stage.

## Supplementary Information

Below is the link to the electronic supplementary material.

Supplementary file1 (DOCX 648 KB)

## Data Availability

Individual data has not been shared due to the higher risk of participant identification associated with the low incidence of HD.
