# Supplementary material for: Feasibility and effects of cognitive training on cognition and psychosocial function in Huntington’s disease: a randomised pilot trial
Source: J Neurol. 2025 Jan 23;272(2):159. doi: 10.1007/s00415-024-12855-7 (PMC11757664; doi:10.1007/s00415-024-12855-7)
Supplement: Supplementary file 1 — Supplementary file1 (DOCX 648 KB) [file 415_2024_12855_MOESM1_ESM.docx]

Article title: Feasibility and effects of cognitive training on cognition and psychosocial function in Huntington’s disease: a randomised pilot trial

Journal name: Journal of Neurology

Author names: Katharine Huynh, Sharna D. Jamadar, Amit Lampit, M. Navyaan Siddiqui, Julie C. Stout, Nellie Georgiou-Karistianis

Corresponding author: Prof Nellie Georgiou-Karistianis
Affiliation: Turner Institute for Brain and Mental Health, 18 Innovation Walk, Monash University, Clayton Victoria 3800, Australia
E-mail: nellie.georgiou-karistianis@monash.edu

Table of Contents

[Cognitive training schedule 2](#_Toc174389250)

[Computerised experimental tasks 4](#_Toc174389251)

[Letter-number task-switching 4](#_Toc174389252)

[Figure S1. Design of the letter-number task switching paradigm. 4](#_Toc174389253)

[Modified Symbol Digits Modalities Test 5](#_Toc174389254)

[Figure S2. Design of the modified Symbol Digits Modalities Test. 6](#_Toc174389255)

[Linear mixed effects (LME) models 7](#_Toc174389256)

[LME models (no covariates) 7](#_Toc174389257)

[LME models with modality as covariate (face-to-face versus remote) 10](#_Toc174389258)

[LME models with experimental task context as covariate (MRI versus computer) 13](#_Toc174389259)

[Effects of individual factors on change in cognitive task performance 15](#_Toc174389260)

[Figure S3. Change in performance on Stroop interference score and task-switching switch accuracy from baseline to follow up. 16](#_Toc174389261)

# **Cognitive training schedule**

Table S1. Selected BrainHQ training exercises

| **Exercise** | **Domain (BrainHQ)** | **Cognitive domain** | **Description** | **Progression** |
| --- | --- | --- | --- | --- |
| **Double decision** | Attention | Processing speed, visuospatial divided attention | User identifies type of car at centre of screen, and location of Route 66 sign in periphery after they are presented simultaneously | Stimuli are presented quicker, signs are presented further from centre of screen, increased similarity of target and distractors, background becomes more complex |
| **Eye for detail** | Brain Speed | Processing speed, visuospatial working memory | Stimuli are presented one after another, and user is required to identify identical stimuli after they disappear | Stimuli are presented more quickly, there is an increased number of stimuli, background becomes more complex |
| **To do list training** | Memory | Verbal (auditory) working memory | User is required to grab items in order, according to an auditory list | Number of items and complexity of instructions increase, instructions are presented more quickly |
| **Mind bender** | Intelligence | Cognitive flexibility, inhibition | User is required to respond according to a different set of rules, depending on type of stimulus e.g., if words, then select smaller number, but if digit, then select larger number | Time to respond decreases, rules become more complex |
| **Juggle factor** | Intelligence | Visuospatial working memory, visuospatial attention | User is required to recall sequence of digits appearing in moving circles in ascending order | Sequence becomes longer and more complex, circles move more quickly and randomly |
| **Divided attention** | Attention | Visuospatial selective attention, inhibition | User is required to determine whether stimuli match or do not match on specific category, e.g., colour | Time to respond decreases, rules increase in complexity e.g., press left if they are same colour and shape |
| **Hawkeye (optional)** | Brain Speed | Processing speed, visuospatial attention | User is required to identify odd one out in array of birds after they are presented simultaneously | Stimuli are presented quicker, stimuli become more visually similar, and background becomes more complex |
| **Mixed signals (optional)** | Attention | Visual and auditory attention, inhibition | User responds only when visual and auditory stimulus match | Time to respond decreases, stimuli become more complex e.g., user hears 'blue' and text is in blue font, but says 'yellow' |

# **Computerised experimental tasks**

## **Letter-number task-switching**


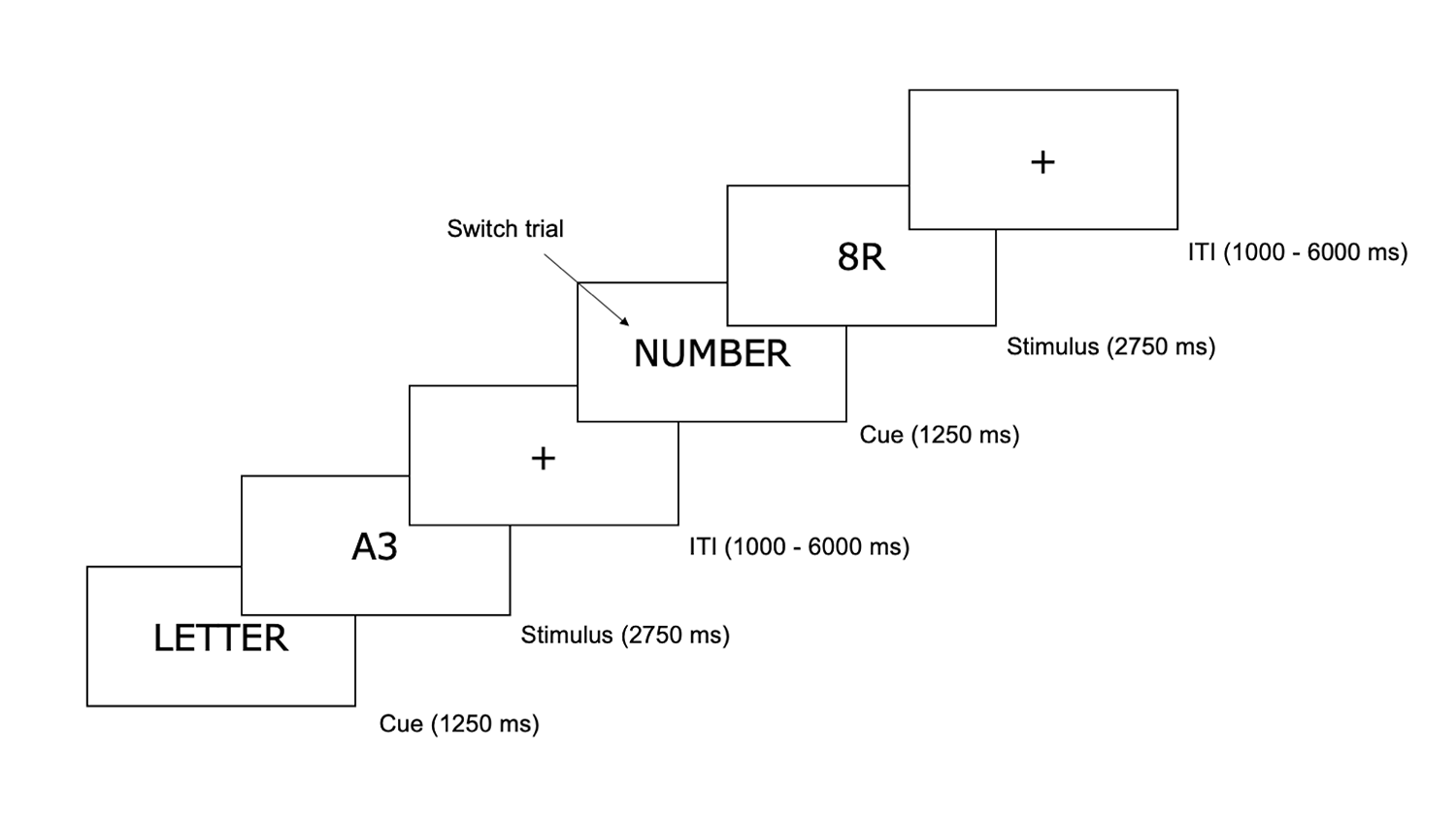


Figure S1. Design of the letter-number task switching paradigm. Abbreviation: ITI = intertrial interval.

The letter-number task switching paradigm involves participants switching randomly between completing either a letter task or a number task (Fig S1). In the letter task, participants are required to classify the letter as vowel (A, E, I, U) or consonant (G, K, M, R). For the number task, participants are required to classify the number as odd (3, 5, 7, 9) or even (2, 4, 6, 8). Participants will use their left and right index fingers to respond. Stimulus-response mapping will be counterbalanced across participants. Participants will be asked to respond as quickly and as accurately as possible. Upon a response, the stimulus will be replaced by a tick (if correct) or cross (if incorrect) for the remaining duration of the trial.

Only incongruent letter–number combinations are presented, i.e., the task-irrelevant character is mapped to a response with the other hand. The switch and repeat trials occur in a pseudorandom sequence that includes no more than four consecutive trials of the same condition (switch, repeat) or repeating stimuli. The intertrial interval (ITI) is 1, 2, 3, 4, 5 or 6 s, with a mean of 3.5 s, during which a central fixation cross is presented. Each participant will complete a total of 72 switch and 72 repeat trials (total 144 trials, 18 mins), across two blocks of 72 trials. Average accuracy and reaction times on switch and repeat trials, and switch costs (difference in accuracy and reaction time between switch and repeat trials) will be calculated.

During the pre-baseline session, participants will be trained on the task on a computer. The practice session will involve one block (24 trials) of each single task (letter or number), and three blocks (24, 32, and 32 trials respectively) of the switch condition (total 136 trials, 16.5 mins). Participants will again be given brief practice on the task on the day of the baseline assessment (eight trials of each single task, and two blocks of 24 trials of the switch task, total 64 trials, 8 mins). On the day of the follow-up assessment, participants will again complete a practice session, i.e., one block (24 trials) of each single task (letter or number), and three blocks (24, 32, and 32 trials respectively) of the switch condition (total 136 trials, 16.5 mins).

## **Modified Symbol Digits Modalities Test**


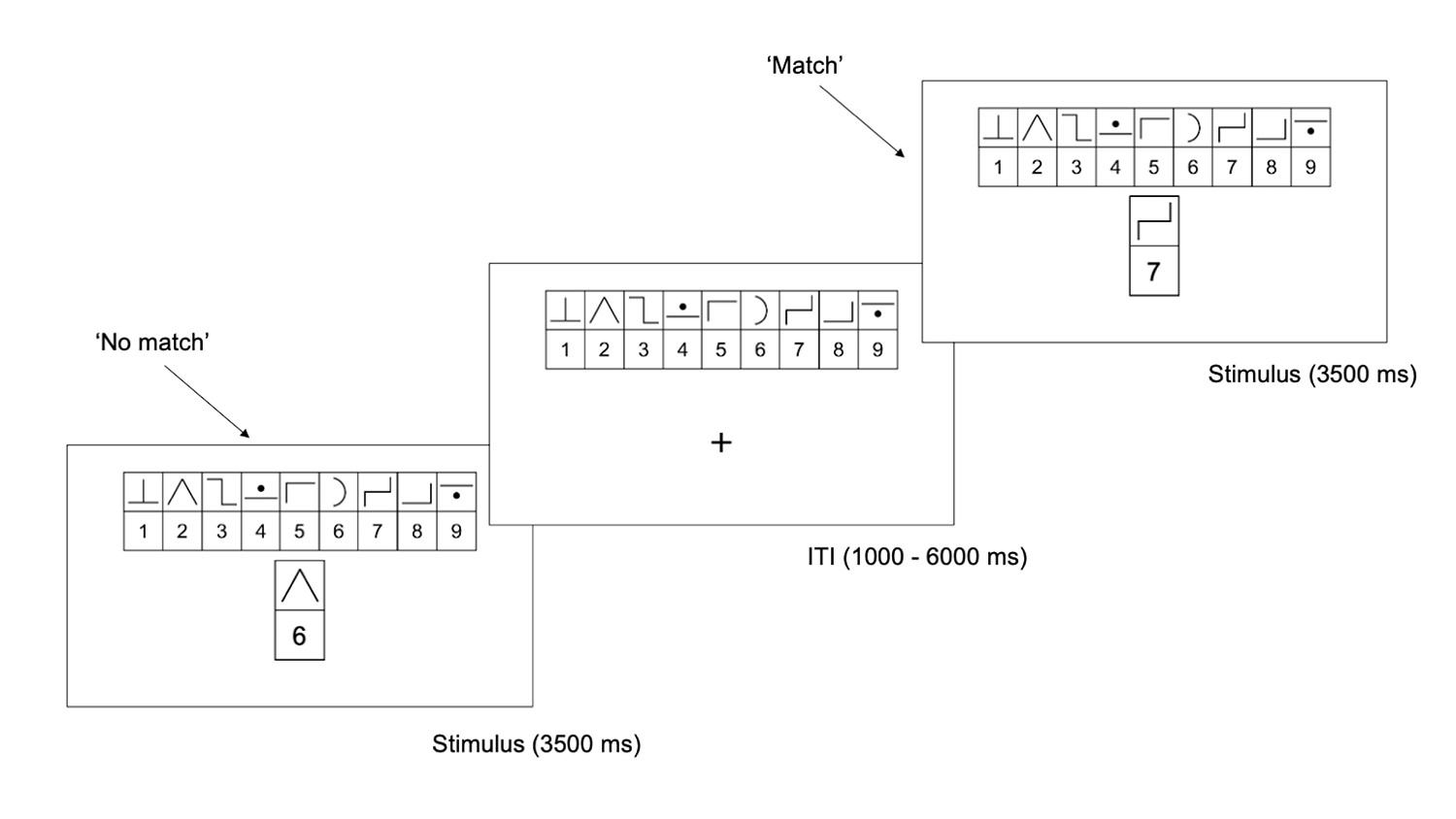


Figure S2. Design of the modified Symbol Digits Modalities Test. Abbreviation: ITI = intertrial interval.

The modified Symbol Digits Modalities Test (SDMT) requires indicating whether a digit-symbol probe matches a digit-symbol pair in a coding table above (Fig S2). Participants will have to indicate whether the probe matches or does not match by using their left or right index fingers. Stimulus-response mapping will be counterbalanced across participants. Participants will be given 3.5 s to respond, and they will be asked to respond as quickly as possible. Upon a response, the symbol-digit probe will be replaced by a tick (if correct) or cross (if incorrect) for the remaining duration of the trial.

Match and no match responses are pseudorandomised, such that there are no more than four consecutive trials of the same condition, or repeating stimuli. The ITI is 1, 2, 3, 4, 5 or 6 s, during which the coding table remains on the screen and a fixation cross is presented in the location of the symbol-digit probe. Participants will complete 72 match and no match trials (total 144 trials, 16.8 mins), across two blocks of 72 trials. Average accuracy and reaction time will be calculated across trials.

During the pre-baseline session, participants will be trained on the task on a computer. The practice session involves two blocks of 10 trials, and one block of 25 trials (total 45 trials, 5.3 mins). Participants will be given practice on the task on the day of the baseline session (45 trials, 5.3 mins). On the day of the follow-up assessment, participants will again complete a practice session, i.e., two blocks of 10 trials, and one block of 25 trials (total 45 trials, 5.3 mins).

# **Linear mixed effects (LME) models**

LME models were fitted using restricted maximum likelihood (REML) method. The following tables are ANOVA tables with F-tests and p-values using Satterthwaite's method.

## **LME models (no covariates)**

Formula: variable ~ 1 + group*timepoint + (1|record_id)

| **SDMT** | **Sum Sq** | **Mean Sq** | **Num DF** | **Den DF** | **F** | **Pr(>F)** |
| --- | --- | --- | --- | --- | --- | --- |
| group | 13.975 | 13.975 | 1 | 26.017 | 1.0844 | 0.30729 |
| timepoint | 34.664 | 34.664 | 1 | 25.046 | 2.6898 | 0.11349 |
| group:timepoint | 54.004 | 54.004 | 1 | 25.046 | 4.1905 | 0.05128 |

| **Digit Span Forwards** | **Sum Sq** | **Mean Sq** | **Num DF** | **Den DF** | **F** | **Pr(>F)** |
| --- | --- | --- | --- | --- | --- | --- |
| group | 0.0161 | 0.0161 | 1 | 26.192 | 0.0121 | 0.9131 |
| timepoint | 1.23576 | 1.23576 | 1 | 25.381 | 0.9306 | 0.3438 |
| group:timepoint | 0.70569 | 0.70569 | 1 | 25.381 | 0.5314 | 0.4727 |

| **Digit Span Backwards** | **Sum Sq** | **Mean Sq** | **Num DF** | **Den DF** | **F** | **Pr(>F)** |
| --- | --- | --- | --- | --- | --- | --- |
| group | 1.22481 | 1.22481 | 1 | 26.124 | 0.9631 | 0.3354 |
| timepoint | 0.0397 | 0.0397 | 1 | 25.302 | 0.0312 | 0.8612 |
| group:timepoint | 0.08329 | 0.08329 | 1 | 25.302 | 0.0655 | 0.8001 |

| **Stroop Color** | **Sum Sq** | **Mean Sq** | **Num DF** | **Den DF** | **F** | **Pr(>F)** |
| --- | --- | --- | --- | --- | --- | --- |
| group | 2.055 | 2.055 | 1 | 26.021 | 0.118 | 0.734 |
| timepoint | 0.169 | 0.169 | 1 | 25.05 | 0.0097 | 0.9222 |
| group:timepoint | 63.37 | 63.37 | 1 | 25.05 | 3.6382 | 0.068 |

| **Stroop Word** | **Sum Sq** | **Mean Sq** | **Num DF** | **Den DF** | **F** | **Pr(>F)** |
| --- | --- | --- | --- | --- | --- | --- |
| group | 0.399 | 0.399 | 1 | 26.032 | 0.0198 | 0.88925 |
| timepoint | 24.206 | 24.206 | 1 | 25.058 | 1.1997 | 0.28379 |
| group:timepoint | 82.076 | 82.076 | 1 | 25.058 | 4.0679 | 0.05454 |

| **Stroop Interference** | **Sum Sq** | **Mean Sq** | **Num DF** | **Den DF** | **F** | **Pr(>F)** |
| --- | --- | --- | --- | --- | --- | --- |
| group | 1.764 | 1.764 | 1 | 26.038 | 0.1749 | 0.679251 |
| timepoint | 0.149 | 0.149 | 1 | 25.068 | 0.0148 | 0.904158 |
| group:timepoint | 117.888 | 117.888 | 1 | 25.068 | 11.6879 | 0.002158 |

| **Trails A time** | **Sum Sq** | **Mean Sq** | **Num DF** | **Den DF** | **F** | **Pr(>F)** |
| --- | --- | --- | --- | --- | --- | --- |
| group | 2.634 | 2.634 | 1 | 26.038 | 0.141 | 0.7103 |
| timepoint | 34.256 | 34.256 | 1 | 25.078 | 1.8343 | 0.1877 |
| group:timepoint | 49.993 | 49.993 | 1 | 25.078 | 2.677 | 0.1143 |

| **Trails B time** | **Sum Sq** | **Mean Sq** | **Num DF** | **Den DF** | **F** | **Pr(>F)** |
| --- | --- | --- | --- | --- | --- | --- |
| group | 23.223 | 23.223 | 1 | 26.024 | 0.1538 | 0.6981 |
| timepoint | 100.51 | 100.51 | 1 | 25.06 | 0.6658 | 0.4222 |
| group:timepoint | 257.053 | 257.053 | 1 | 25.06 | 1.7028 | 0.2038 |

| **Trails Be Score** | **Sum Sq** | **Mean Sq** | **Num DF** | **Den DF** | **F** | **Pr(>F)** |
| --- | --- | --- | --- | --- | --- | --- |
| group | 0.16392 | 0.16392 | 1 | 26.051 | 0.2377 | 0.62992 |
| timepoint | 0.01519 | 0.01519 | 1 | 25.126 | 0.022 | 0.88318 |
| group:timepoint | 2.04997 | 2.04997 | 1 | 25.126 | 2.9733 | 0.09693 |

| **Spatial Span forwards** | **Sum Sq** | **Mean Sq** | **Num DF** | **Den DF** | **F** | **Pr(>F)** |
| --- | --- | --- | --- | --- | --- | --- |
| group | 0.20291 | 0.20291 | 1 | 16.357 | 0.182 | 0.6752 |
| timepoint | 1.10243 | 1.10243 | 1 | 15.586 | 0.989 | 0.3352 |
| group:timepoint | 2.24772 | 2.24772 | 1 | 15.586 | 2.0164 | 0.1753 |

| **Spatial Span backwards** | **Sum Sq** | **Mean Sq** | **Num DF** | **Den DF** | **F** | **Pr(>F)** |
| --- | --- | --- | --- | --- | --- | --- |
| group | 1.63419 | 1.63419 | 1 | 16.338 | 0.7372 | 0.403 |
| timepoint | 0.21584 | 0.21584 | 1 | 15.75 | 0.0974 | 0.7591 |
| group:timepoint | 0.09216 | 0.09216 | 1 | 15.75 | 0.0416 | 0.841 |

| **TSWT switch accuracy** | **Sum Sq** | **Mean Sq** | **Num DF** | **Den DF** | **F** | **Pr(>F)** |
| --- | --- | --- | --- | --- | --- | --- |
| group | 6.87 | 6.87 | 1 | 21.42 | 0.9627 | 0.33746 |
| timepoint | 0.02 | 0.02 | 1 | 20.937 | 0.0028 | 0.95853 |
| group:timepoint | 52.815 | 52.815 | 1 | 20.937 | 7.401 | 0.01284 |

| **TSWT switch RT** | **Sum Sq** | **Mean Sq** | **Num DF** | **Den DF** | **F** | **Pr(>F)** |
| --- | --- | --- | --- | --- | --- | --- |
| group | 3481.4 | 3481.4 | 1 | 21.093 | 0.2552 | 0.6187 |
| timepoint | 9951.8 | 9951.8 | 1 | 20.336 | 0.7294 | 0.403 |
| group:timepoint | 10214.8 | 10214.8 | 1 | 20.336 | 0.7487 | 0.397 |

| **TSWT repeat accuracy** | **Sum Sq** | **Mean Sq** | **Num DF** | **Den DF** | **F** | **Pr(>F)** |
| --- | --- | --- | --- | --- | --- | --- |
| group | 2.41751 | 2.41751 | 1 | 21.364 | 0.6483 | 0.4296 |
| timepoint | 2.09914 | 2.09914 | 1 | 20.774 | 0.5629 | 0.4615 |
| group:timepoint | 0.54656 | 0.54656 | 1 | 20.774 | 0.1466 | 0.7057 |

| **TSWT repeat RT** | **Sum Sq** | **Mean Sq** | **Num DF** | **Den DF** | **F** | **Pr(>F)** |
| --- | --- | --- | --- | --- | --- | --- |
| group | 168.4 | 168.4 | 1 | 21.145 | 0.0148 | 0.9044 |
| timepoint | 2079.9 | 2079.9 | 1 | 20.351 | 0.1826 | 0.6736 |
| group:timepoint | 8185.2 | 8185.2 | 1 | 20.351 | 0.7188 | 0.4064 |

| **TSWT switch cost accuracy** | **Sum Sq** | **Mean Sq** | **Num DF** | **Den DF** | **F** | **Pr(>F)** |
| --- | --- | --- | --- | --- | --- | --- |
| group | 2.261 | 2.261 | 1 | 41 | 0.2965 | 0.589047 |
| timepoint | 3.06 | 3.06 | 1 | 41 | 0.4013 | 0.529928 |
| group:timepoint | 67.406 | 67.406 | 1 | 41 | 8.8399 | 0.004917 |

| **TSWT switch cost RT** | **Sum Sq** | **Mean Sq** | **Num DF** | **Den DF** | **F** | **Pr(>F)** |
| --- | --- | --- | --- | --- | --- | --- |
| group | 11737.3 | 11737.3 | 1 | 41 | 2.9835 | 0.09164 |
| timepoint | 2525.7 | 2525.7 | 1 | 41 | 0.642 | 0.42761 |
| group:timepoint | 38.3 | 38.3 | 1 | 41 | 0.0097 | 0.92187 |

| **Modified SDMT accuracy** | **Sum Sq** | **Mean Sq** | **Num DF** | **Den DF** | **F** | **Pr(>F)** |
| --- | --- | --- | --- | --- | --- | --- |
| group | 8.155 | 8.155 | 1 | 25.233 | 0.374 | 0.54628 |
| timepoint | 78.609 | 78.609 | 1 | 24.491 | 3.6057 | 0.06943 |
| group:timepoint | 4.334 | 4.334 | 1 | 24.491 | 0.1988 | 0.65963 |

| **Modified SDMT RT** | **Sum Sq** | **Mean Sq** | **Num DF** | **Den DF** | **F** | **Pr(>F)** |
| --- | --- | --- | --- | --- | --- | --- |
| group | 6096.9 | 6096.9 | 1 | 25.05 | 1.0904 | 0.30636 |
| timepoint | 1361.1 | 1361.1 | 1 | 24.09 | 0.2434 | 0.62621 |
| group:timepoint | 20670.4 | 20670.4 | 1 | 24.09 | 3.6967 | 0.06642 |

| **CDS** | **Sum Sq** | **Mean Sq** | **Num DF** | **Den DF** | **F** | **Pr(>F)** |
| --- | --- | --- | --- | --- | --- | --- |
| group | 16.2 | 16.2 | 1 | 26 | 0.2046 | 0.6548 |
| timepoint | 1684.57 | 1684.57 | 1 | 26 | 21.2773 | 9.34E-05 |
| group:timepoint | 174.14 | 174.14 | 1 | 26 | 2.1995 | 0.1501 |

| **HADS total score** | **Sum Sq** | **Mean Sq** | **Num DF** | **Den DF** | **F** | **Pr(>F)** |
| --- | --- | --- | --- | --- | --- | --- |
| group | 9.3971 | 9.3971 | 1 | 26 | 1.5939 | 0.218 |
| timepoint | 0.1465 | 0.1465 | 1 | 26 | 0.0249 | 0.876 |
| group:timepoint | 2.6465 | 2.6465 | 1 | 26 | 0.4489 | 0.5088 |

| **HD-PRO-TRIAD total score** | **Sum Sq** | **Mean Sq** | **Num DF** | **Den DF** | **F** | **Pr(>F)** |
| --- | --- | --- | --- | --- | --- | --- |
| group | 0.48186 | 0.48186 | 1 | 26 | 1.1387 | 0.29574 |
| timepoint | 1.5726 | 1.5726 | 1 | 26 | 3.7162 | 0.06488 |
| group:timepoint | 0.74894 | 0.74894 | 1 | 26 | 1.7698 | 0.19495 |

## **LME models with modality as covariate (face-to-face versus remote)**

Formula: variable ~ 1 + group*timepoint + mode + (1|record_id)

| **SDMT** | **Sum Sq** | **Mean Sq** | **Num DF** | **Den DF** | **F** | **Pr(>F)** |
| --- | --- | --- | --- | --- | --- | --- |
| group | 45.05 | 45.05 | 1 | 25.061 | 3.4976 | 0.073182 |
| timepoint | 34.176 | 34.176 | 1 | 25.083 | 2.6533 | 0.11583 |
| mode | 140.719 | 140.719 | 1 | 25.036 | 10.9251 | 0.002864 |
| group:timepoint | 53.396 | 53.396 | 1 | 25.083 | 4.1455 | 0.052434 |

| **Digit Span Forwards** | **Sum Sq** | **Mean Sq** | **Num DF** | **Den DF** | **F** | **Pr(>F)** |
| --- | --- | --- | --- | --- | --- | --- |
| group | 0.0501 | 0.0501 | 1 | 25.246 | 0.0377 | 0.8476 |
| timepoint | 1.22337 | 1.22337 | 1 | 25.353 | 0.9209 | 0.3463 |
| mode | 0.24261 | 0.24261 | 1 | 25.142 | 0.1826 | 0.6728 |
| group:timepoint | 0.71467 | 0.71467 | 1 | 25.353 | 0.538 | 0.47 |

| **Digit Span Backwards** | **Sum Sq** | **Mean Sq** | **Num DF** | **Den DF** | **F** | **Pr(>F)** |
| --- | --- | --- | --- | --- | --- | --- |
| group | 1.88807 | 1.88807 | 1 | 25.228 | 1.4858 | 0.2341 |
| timepoint | 0.04614 | 0.04614 | 1 | 25.333 | 0.0363 | 0.8504 |
| mode | 1.87133 | 1.87133 | 1 | 25.127 | 1.4727 | 0.2362 |
| group:timepoint | 0.07455 | 0.07455 | 1 | 25.333 | 0.0587 | 0.8106 |

| **Stroop Colour** | **Sum Sq** | **Mean Sq** | **Num DF** | **Den DF** | **F** | **Pr(>F)** |
| --- | --- | --- | --- | --- | --- | --- |
| Group | 15.696 | 15.696 | 1 | 24.99 | 0.9003 | 0.351799 |
| Timepoint | 0.247 | 0.247 | 1 | 25.01 | 0.0142 | 0.906155 |
| Mode | 136.69 | 136.69 | 1 | 24.967 | 7.8401 | 0.009716 |
| group:timepoint | 64.751 | 64.751 | 1 | 25.01 | 3.7139 | 0.065395 |

| **Stroop Word** | **Sum Sq** | **Mean Sq** | **Num DF** | **Den DF** | **F** | **Pr(>F)** |
| --- | --- | --- | --- | --- | --- | --- |
| group | 10.063 | 10.063 | 1 | 25.047 | 0.4987 | 0.48657 |
| timepoint | 24.82 | 24.82 | 1 | 25.064 | 1.2301 | 0.27791 |
| mode | 148.821 | 148.821 | 1 | 25.026 | 7.3759 | 0.01181 |
| group:timepoint | 83.208 | 83.208 | 1 | 25.064 | 4.124 | 0.05302 |

| **Stroop Interference** | **Sum Sq** | **Mean Sq** | **Num DF** | **Den DF** | **F** | **Pr(>F)** |
| --- | --- | --- | --- | --- | --- | --- |
| group | 10.224 | 10.224 | 1 | 25.055 | 1.0137 | 0.323646 |
| timepoint | 0.188 | 0.188 | 1 | 25.075 | 0.0186 | 0.89262 |
| mode | 73.739 | 73.739 | 1 | 25.031 | 7.3108 | 0.012142 |
| group:timepoint | 118.922 | 118.922 | 1 | 25.075 | 11.7905 | 0.002078 |

| **Trails A time** | **Sum Sq** | **Mean Sq** | **Num DF** | **Den DF** | **F** | **Pr(>F)** |
| --- | --- | --- | --- | --- | --- | --- |
| group | 0.073 | 0.073 | 1 | 25.065 | 0.0039 | 0.9505 |
| timepoint | 33.83 | 33.83 | 1 | 25.088 | 1.8119 | 0.1903 |
| mode | 44.581 | 44.581 | 1 | 25.039 | 2.3877 | 0.1348 |
| group:timepoint | 49.478 | 49.478 | 1 | 25.088 | 2.65 | 0.116 |

| **Trails B time** | **Sum Sq** | **Mean Sq** | **Num DF** | **Den DF** | **F** | **Pr(>F)** |
| --- | --- | --- | --- | --- | --- | --- |
| group | 2.331 | 2.331 | 1 | 25.05 | 0.0154 | 0.9021 |
| timepoint | 102.193 | 102.193 | 1 | 25.07 | 0.6771 | 0.4183 |
| mode | 255.383 | 255.383 | 1 | 25.027 | 1.6921 | 0.2052 |
| group:timepoint | 254.375 | 254.375 | 1 | 25.07 | 1.6854 | 0.206 |

| **Trails Be score** | **Sum Sq** | **Mean Sq** | **Num DF** | **Den DF** | **F** | **Pr(>F)** |
| --- | --- | --- | --- | --- | --- | --- |
| group | 0.04058 | 0.04058 | 1 | 25.1 | 0.0589 | 0.81026 |
| timepoint | 0.01707 | 0.01707 | 1 | 25.141 | 0.0248 | 0.87621 |
| mode | 0.93388 | 0.93388 | 1 | 25.055 | 1.355 | 0.25537 |
| group:timepoint | 2.0288 | 2.0288 | 1 | 25.141 | 2.9436 | 0.09852 |

| **TSWT switch accuracy** | **Sum Sq** | **Mean Sq** | **Num DF** | **Den DF** | **F** | **Pr(>F)** |
| --- | --- | --- | --- | --- | --- | --- |
| group | 9.68 | 9.68 | 1 | 20.503 | 1.3542 | 0.2579 |
| timepoint | 0.04 | 0.04 | 1 | 20.88 | 0.0056 | 0.9413 |
| mode | 7.519 | 7.519 | 1 | 20.174 | 1.0519 | 0.31721 |
| group:timepoint | 53.651 | 53.651 | 1 | 20.88 | 7.5057 | 0.01232 |

| **TSWT switch RT** | **Sum Sq** | **Mean Sq** | **Num DF** | **Den DF** | **F** | **Pr(>F)** |
| --- | --- | --- | --- | --- | --- | --- |
| group | 11363 | 11363 | 1 | 20.293 | 0.8357 | 0.37137 |
| timepoint | 10542 | 10542 | 1 | 20.477 | 0.7753 | 0.38881 |
| mode | 56998 | 56998 | 1 | 20.08 | 4.1919 | 0.05393 |
| group:timepoint | 10812 | 10812 | 1 | 20.477 | 0.7952 | 0.38289 |

| **TSWT repeat accuracy** | **Sum Sq** | **Mean Sq** | **Num DF** | **Den DF** | **F** | **Pr(>F)** |
| --- | --- | --- | --- | --- | --- | --- |
| group | 3.3023 | 3.3023 | 1 | 20.485 | 0.8858 | 0.3576 |
| timepoint | 2.0066 | 2.0066 | 1 | 20.768 | 0.5383 | 0.4714 |
| mode | 2.4994 | 2.4994 | 1 | 20.203 | 0.6705 | 0.4224 |
| group:timepoint | 0.5001 | 0.5001 | 1 | 20.768 | 0.1342 | 0.7179 |

| **TSWT repeat RT** | **Sum Sq** | **Mean Sq** | **Num DF** | **Den DF** | **F** | **Pr(>F)** |
| --- | --- | --- | --- | --- | --- | --- |
| group | 2820 | 2820 | 1 | 20.301 | 0.2482 | 0.62371 |
| timepoint | 2325 | 2325 | 1 | 20.453 | 0.2046 | 0.65578 |
| mode | 44881 | 44881 | 1 | 20.116 | 3.9505 | 0.06064 |
| group:timepoint | 8666 | 8666 | 1 | 20.453 | 0.7628 | 0.39258 |

| **TSWT switch cost accuracy** | **Sum Sq** | **Mean Sq** | **Num DF** | **Den DF** | **F** | **Pr(>F)** |
| --- | --- | --- | --- | --- | --- | --- |
| group | 3.23 | 3.23 | 1 | 40 | 0.4167 | 0.522299 |
| timepoint | 3.195 | 3.195 | 1 | 40 | 0.4121 | 0.524571 |
| mode | 2.539 | 2.539 | 1 | 40 | 0.3276 | 0.5703 |
| group:timepoint | 68.001 | 68.001 | 1 | 40 | 8.7717 | 0.005127 |

| **TSWT switch cost RT** | **Sum Sq** | **Mean Sq** | **Num DF** | **Den DF** | **F** | **Pr(>F)** |
| --- | --- | --- | --- | --- | --- | --- |
| group | 11648.8 | 11648.8 | 1 | 40 | 2.8903 | 0.09688 |
| timepoint | 2546.6 | 2546.6 | 1 | 40 | 0.6318 | 0.43137 |
| mode | 84.1 | 84.1 | 1 | 40 | 0.0209 | 0.88585 |
| group:timepoint | 41.1 | 41.1 | 1 | 40 | 0.0102 | 0.92009 |

| **Modified SDMT accuracy** | **Sum Sq** | **Mean Sq** | **Num DF** | **Den DF** | **F** | **Pr(>F)** |
| --- | --- | --- | --- | --- | --- | --- |
| group | 9.84 | 9.84 | 1 | 24.304 | 0.4514 | 0.50802 |
| timepoint | 79.195 | 79.195 | 1 | 24.481 | 3.6329 | 0.06845 |
| mode | 4.92 | 4.92 | 1 | 24.134 | 0.2257 | 0.63901 |
| group:timepoint | 4.477 | 4.477 | 1 | 24.481 | 0.2054 | 0.6544 |

| **Modified SDMT RT** | **Sum Sq** | **Mean Sq** | **Num DF** | **Den DF** | **F** | **Pr(>F)** |
| --- | --- | --- | --- | --- | --- | --- |
| group | 9648.7 | 9648.7 | 1 | 24.053 | 1.7252 | 0.20142 |
| timepoint | 1417 | 1417 | 1 | 24.081 | 0.2533 | 0.6193 |
| mode | 14020.4 | 14020.4 | 1 | 24.02 | 2.5068 | 0.12643 |
| group:timepoint | 20886.8 | 20886.8 | 1 | 24.081 | 3.7345 | 0.06514 |

| **CDS score** | **Sum Sq** | **Mean Sq** | **Num DF** | **Den DF** | **F** | **Pr(>F)** |
| --- | --- | --- | --- | --- | --- | --- |
| group | 64.27 | 64.27 | 1 | 25 | 0.8117 | 0.3762 |
| timepoint | 1684.57 | 1684.57 | 1 | 26 | 21.2773 | 9.34E-05 |
| mode | 354.74 | 354.74 | 1 | 25 | 4.4806 | 0.04441 |
| group:timepoint | 174.14 | 174.14 | 1 | 26 | 2.1995 | 0.15007 |

| **HADS score** | **Sum Sq** | **Mean Sq** | **Num DF** | **Den DF** | **F** | **Pr(>F)** |
| --- | --- | --- | --- | --- | --- | --- |
| group | 9.0122 | 9.0122 | 1 | 25 | 1.5287 | 0.2278 |
| timepoint | 0.1465 | 0.1465 | 1 | 26 | 0.0249 | 0.876 |
| mode | 0.0811 | 0.0811 | 1 | 25 | 0.0138 | 0.9076 |
| group:timepoint | 2.6465 | 2.6465 | 1 | 26 | 0.4489 | 0.5088 |

| **HD-PRO-TRIAD score** | **Sum Sq** | **Mean Sq** | **Num DF** | **Den DF** | **F** | **Pr(>F)** |
| --- | --- | --- | --- | --- | --- | --- |
| group | 0.84802 | 0.84802 | 1 | 25 | 2.004 | 0.16922 |
| timepoint | 1.5726 | 1.5726 | 1 | 26 | 3.7162 | 0.06488 |
| mode | 1.16247 | 1.16247 | 1 | 25 | 2.7471 | 0.10993 |
| group:timepoint | 0.74894 | 0.74894 | 1 | 26 | 1.7698 | 0.19495 |

## **LME models with experimental task context as covariate (MRI versus computer)**

Formula: variable ~ 1 + group*timepoint + mri + (1|record_id)

| **TSWT switch accuracy** | **Sum Sq** | **Mean Sq** | **Num DF** | **Den DF** | **F** | **Pr(>F)** |
| --- | --- | --- | --- | --- | --- | --- |
| group | 8.819 | 8.819 | 1 | 20.436 | 1.2338 | 0.27957 |
| timepoint | 0.042 | 0.042 | 1 | 20.884 | 0.0059 | 0.93959 |
| mri | 9.7 | 9.7 | 1 | 20.141 | 1.357 | 0.25766 |
| group:timepoint | 53.748 | 53.748 | 1 | 20.884 | 7.5192 | 0.01225 |

| **TSWT switch RT** | **Sum Sq** | **Mean Sq** | **Num DF** | **Den DF** | **F** | **Pr(>F)** |
| --- | --- | --- | --- | --- | --- | --- |
| group | 5796.7 | 5796.7 | 1 | 20.209 | 0.4258 | 0.5214 |
| timepoint | 10396 | 10396 | 1 | 20.416 | 0.7637 | 0.3923 |
| mri | 25917.5 | 25917.5 | 1 | 20.032 | 1.9039 | 0.1828 |
| group:timepoint | 10664.8 | 10664.8 | 1 | 20.416 | 0.7835 | 0.3864 |

| **TSWT repeat accuracy** | **Sum Sq** | **Mean Sq** | **Num DF** | **Den DF** | **F** | **Pr(>F)** |
| --- | --- | --- | --- | --- | --- | --- |
| group | 2.8677 | 2.8677 | 1 | 20.428 | 0.7692 | 0.3906 |
| timepoint | 2.0134 | 2.0134 | 1 | 20.768 | 0.5401 | 0.4706 |
| mri | 2.3253 | 2.3253 | 1 | 20.178 | 0.6237 | 0.4388 |
| group:timepoint | 0.5035 | 0.5035 | 1 | 20.768 | 0.1351 | 0.717 |

| **TSWT repeat RT** | **Sum Sq** | **Mean Sq** | **Num DF** | **Den DF** | **F** | **Pr(>F)** |
| --- | --- | --- | --- | --- | --- | --- |
| group | 781.1 | 781.1 | 1 | 20.232 | 0.0687 | 0.7959 |
| timepoint | 2258.6 | 2258.6 | 1 | 20.406 | 0.1986 | 0.6605 |
| mri | 21546.6 | 21546.6 | 1 | 20.078 | 1.895 | 0.1838 |
| group:timepoint | 8536.2 | 8536.2 | 1 | 20.406 | 0.7507 | 0.3963 |

| **TSWT switch cost accuracy** | **Sum Sq** | **Mean Sq** | **Num DF** | **Den DF** | **F** | **Pr(>F)** |
| --- | --- | --- | --- | --- | --- | --- |
| group | 3.1 | 3.1 | 1 | 40 | 0.4033 | 0.529023 |
| timepoint | 3.244 | 3.244 | 1 | 40 | 0.422 | 0.519654 |
| mri | 5.135 | 5.135 | 1 | 40 | 0.668 | 0.418595 |
| group:timepoint | 68.231 | 68.231 | 1 | 40 | 8.8756 | 0.004894 |

| **TSWT switch cost RT** | **Sum Sq** | **Mean Sq** | **Num DF** | **Den DF** | **F** | **Pr(>F)** |
| --- | --- | --- | --- | --- | --- | --- |
| group | 11484.2 | 11484.2 | 1 | 40 | 2.8482 | 0.09926 |
| timepoint | 2516.2 | 2516.2 | 1 | 40 | 0.624 | 0.43421 |
| mri | 12.4 | 12.4 | 1 | 40 | 0.0031 | 0.95601 |
| group:timepoint | 37.3 | 37.3 | 1 | 40 | 0.0092 | 0.92387 |

| **Modified SDMT accuracy** | **Sum Sq** | **Mean Sq** | **Num DF** | **Den DF** | **F** | **Pr(>F)** |
| --- | --- | --- | --- | --- | --- | --- |
| group | 15.093 | 15.093 | 1 | 24.356 | 0.6929 | 0.41328 |
| timepoint | 80.232 | 80.232 | 1 | 24.519 | 3.683 | 0.06668 |
| mri | 35.528 | 35.528 | 1 | 24.223 | 1.6309 | 0.21369 |
| group:timepoint | 4.723 | 4.723 | 1 | 24.519 | 0.2168 | 0.6456 |

| **Modified SDMT RT** | **Sum Sq** | **Mean Sq** | **Num DF** | **Den DF** | **F** | **Pr(>F)** |
| --- | --- | --- | --- | --- | --- | --- |
| group | 11176.4 | 11176.4 | 1 | 24.051 | 1.998 | 0.17032 |
| timepoint | 1443.4 | 1443.4 | 1 | 24.076 | 0.258 | 0.6161 |
| mri | 21022.5 | 21022.5 | 1 | 24.025 | 3.7582 | 0.06438 |
| group:timepoint | 20988.4 | 20988.4 | 1 | 24.076 | 3.7521 | 0.06456 |

# **Effects of individual factors on change in cognitive task performance**

Individual-level plots revealed heterogeneity in change on cognitive tasks from baseline to follow up, particularly in the CCT group. Qualitatively, factors such as disease stage, age, and CAG length did not appear to explain this heterogeneity (Fig S3).


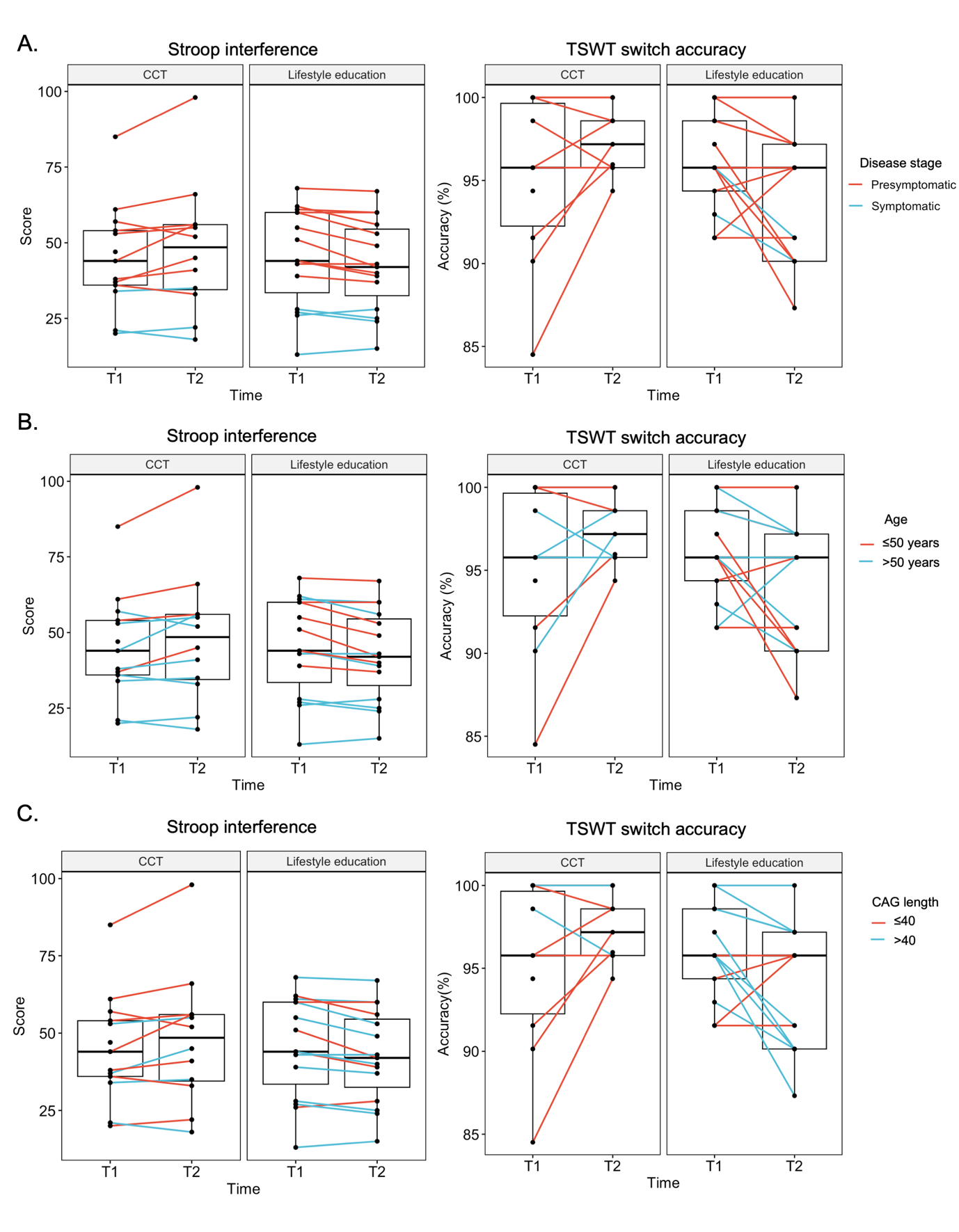


Figure S3. Change in performance on Stroop interference score and task-switching switch accuracy from baseline to follow up. Higher scores represent better performance. Lines are colour-coded based on A) disease stage, B) age, and C) CAG repeat length. Abbreviations: CCT = computerised cognitive training, TSWT = task switching, T1 = baseline, T2 = follow up.
